# Oxidative stress and radioiodine treatment of differentiated thyroid cancer

**DOI:** 10.1038/s41598-021-96637-5

**Published:** 2021-08-24

**Authors:** Angelika Buczyńska, Iwona Sidorkiewicz, Mariusz Rogucki, Katarzyna Siewko, Agnieszka Adamska, Maria Kościuszko, Katarzyna Maliszewska, Gabryela Kozłowska, Piotr Szumowski, Janusz Myśliwiec, Janusz Dzięcioł, Adam Krętowski, Anna Popławska-Kita

**Affiliations:** 1 Clinical Research Centre, Medical University of Bialystok, 15-276 Bialystok, Poland; 2 Department of Endocrinology, Diabetology and Internal Medicine, Medical University of Bialystok, 15-276 Bialystok, Poland; 3 Department of Nuclear Medicine, Medical University of Bialystok, 15-276 Bialystok, Poland; 4 Department of Human Anatomy, Medical University of Bialystok, 15-276 Bialystok, Poland

**Keywords:** Endocrine system and metabolic diseases, Cancer, Radiotherapy

## Abstract

It is hypothesized that the oxidative stress level in thyroid cancer patients is additionally upregulated by radioactive iodine (RAI) treatment, that may exert an important impact on future health concerns. In our study, we evaluated the oxidative stress level changes using the measurement of malondialdehyde (MDA) concentration in patients with differentiated thyroid cancer (DTC) undergoing RAI treatment. Considering the results obtained in the study group, the serum levels of MDA in DTC patients were significantly higher compared to the healthy subjects (*p* < 0.05). The MDA concentration was significantly higher on the third day after RAI (*p* < 0.001) and significantly lower one year after RAI (*p* < 0.05) in DTC patients compared to the baseline concentration. Moreover, the redox stabilization after RAI treatment in patients with DTC during a year-long observation was demonstrated. Accordingly, an increased oxidative stress impact on the related biochemical parameters reflecting the health conditions of the DTC patients was determined. Our study showed that increased oxidative stress reflected by MDA measurements in DTC patients is further enhanced by RAI, but this effect is no longer observed one year after the therapy.

## Introduction

Thyroid cancer is the fifth most frequently occurring type of malignancies, with an estimated number of > 62,000 new cases diagnosed every year^1^. Women are at particular risk for this cancer, with 22.2/100,000 individuals seriously affected every year^2^. Among thyroid follicular tumorigenesis, the largest group includes differentiated carcinomas (approximately 90% of cases), with papillary cancer constituting the vast majority^1^. The most crucial role in the diagnosis and treatment of this malignancy is played by the biopsy, with subsequent thyroid resection^3^. After surgery, radioactive iodine (RAI) treatment is recommended to eradicate potential residual disease and improve prognosis^4,5^. Nevertheless, the increased oxidative stress level observed during cancer metastasis, which promotes transformation with the proliferation of cancer cells, could be additionally upregulated by RAI^5,6^. Despite significant progress in relevant diagnostics tools, thyroid cancer is still a significant clinical problem^7^. The literature data underline the increasing incidence of thyroid cancer overdiagnoses and overtreatment^7,8^. Therefore, new screening biomarkers are constantly being evaluated, not only under the hypothesis of assessing the risk of cancer occurrence, but also to evaluate the effectiveness of undergoing such therapy and the risk of occurrence for late-stage side effects^9^. Malondialdehyde (MDA) is the main naturally occurring product of lipid peroxidation. Accordingly, MDA is a well-established marker for screening and monitoring the oxidation stress level. The MDA measurement was also previously established as a biomarker of oxidative stress and chemotherapy response marker in patients with primary ocular carcinoma^10^. Moreover, the tumor-related cytotoxicity promotion and inhibitory impact on antioxidant enzymes activity can be analyzed by MDA measurement^6–9^. Thus, the aim of this study was to evaluate the MDA concentration in DTC patients undergoing RAI treatment and its correlation with thyroid hormones and lipid profile.

## Results

### Biochemical results

At baseline (V1), the groups did not differ significantly in terms of triglyceride (TG), HDL, total cholesterol (CHOL), and glucose levels (all *p* > 0.05). MDA concentration was higher in DTC group when compared to healthy individuals (*p* < 0.001). The TSH, fT3 and fT4 concentrations were significantly lower among the DTC patients compared to the healthy subjects (*p* < 0.05; *p* < 0.01, and *p* < 0.05; respectively). However, the thyroglobulin (TGB), LDL and CRP concentrations were increased compared to those obtained from the control group (*p* < 0.001; *p* < 0.05 and *p* < 0.001, respectively) (Table 1).

**Table 1:** Comparison of the biochemical parameters between the control and study groups (median and lower and upper quartiles).

	Units	Control group	DTC group			*p*-value (baseline DTC vs. control group)	*p*-value (DTC between visits)
			Visit 1	Visit 2	Visit 3		
MDA	(µM)	1.61 (0.46; 2.12)	3.24 (2.02; 4.88)–9.57)	4.24 (2.94; 5.40)	1.10 (0.47; 4.32)	< 0.001	< 0.001
TSH	(µIU/mL)	1.58 (1.22; 2.19)	0.51 (0.54;2.30)	8.25 (5.13; 26.85)	0.06 (0.02; 0.31)	< 0.05	< 0.001
fT3	(pg/mL)	2.90 (2.50; 3.38)	2.50 (2.21; 2.78–)	2.19 (1.86; 2.40)	2.68 (2.39; 3.03)	< 0.01	< 0.05
fT4	(ng/dL)	1.17 (1.09; 1.35)	1.29 (1.08; 1.46)	1.05 (0.83; 1.33)	1.29 (1.19; 1.41)	< 0.05	< 0.01
TGB	(ng/ml)	0.04 (0.04; 0.05)	10.3 (6.45; 10.70)	2.45 (0.57; 9.78)	0.04 (0.04; 0.04)	< 0.05	< 0.05
TGBAb	(IU/mL)	3.47 (2.43; 7.28)	31.20 (1.36; 7.31))	3.33 (1.64; 6.27)	2.09 (0.74; 7.88)	< 0.05	NS
CRP	(mg/L)	0.48 (0.18; 1.76)	1.30 (0.7;3.30)	1.30 (0.60; 3.05)	1.50 (1.00; 2.80)	< 0.05	NS
TG	(mg/dL)	89.00 (63.00; 139.50)	85.00 (66.5; 126.00)	95.00 (71.25; 149.50)	94.00 (71.00; 129.00)	NS	NS
LDL	(mg/dL)	98.00 (78.8; 122.3)	127.50 (107.3; 151.00)	123.00 (101.00; 168.00)	122.00 (99.00; 164.00)	< 0.05	NS
HDL	(mg/dL)	52.62 (43.87; 64.38)	52.00 (46; 76)	52.00 (45.25; 64.00)	52.00 (44.00; 62.5)	NS	NS
CHOL	(mg/dL)	180.00 (169.50; 219.50)	198.00 (158.50–253.50)	188.00 (161.00; 247.00)	192.00 (153.00; 221.5)	NS	NS
Glucose	(mg/dL)	84.00 (79.75; 98.75)	93.50 (87.00–101.30)	94.00 (86.00; 102.00)	91.00 (86.00; 102.80)	NS	NS

Comparison between the study and control groups was performed using a Mann–Whitney *U* test, and differences between visits among DTC patients were evaluated using a Friedman’s test.

*CHOL* cholesterol, *CRP* C-reactive protein, *DTC* differentiated thyroid cancer, *fT3* free triiodothyronine, *fT4* free thyroxine, *HDL* high-density lipoprotein, *LDL* low-density lipoprotein, *MDA* malondialdehyde, *NS* not significant, *TG* triglyceride, *TGB* thyroglobulin, *TGBAb* thyroglobulin antibodies, *TSH* thyroid-stimulating hormone.

**Figure 1:**
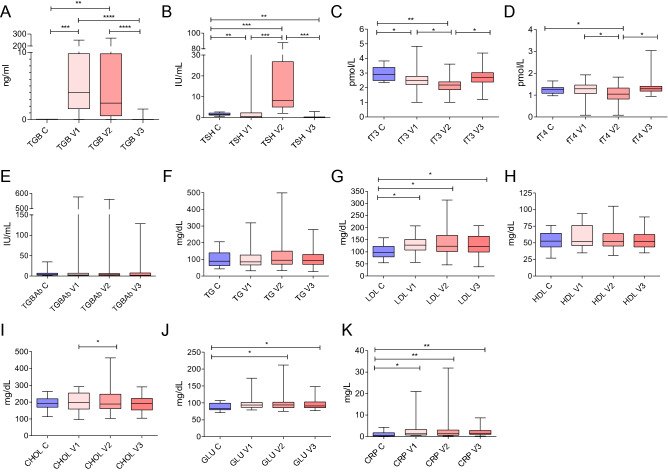
The parameter profiles measured during the study: (**A**) thyroglobulin (TGB); (**B**) thyroid-stimulating hormone (TSH); (**C**) free triiodothyronine (fT3); (**D**) free thyroxine (fT4); (**E**) thyroglobulin antibodies (TGBAb); (**F)** triglyceride (TG); (**G**) low-density lipoprotein (LDL); (**H**) high-density lipoprotein (HDL); (**I**) total cholesterol (CHOL); (**J**) glucose (GLU); (**K**) C-reactive protein (CRP). *C* control group; *V* visit. Asterisks indicate significant differences (*p < 0.05; **p < 0.01; ***p < 0.001; ****p < 0.0001) compared with corresponding control.

**Figure 2:**
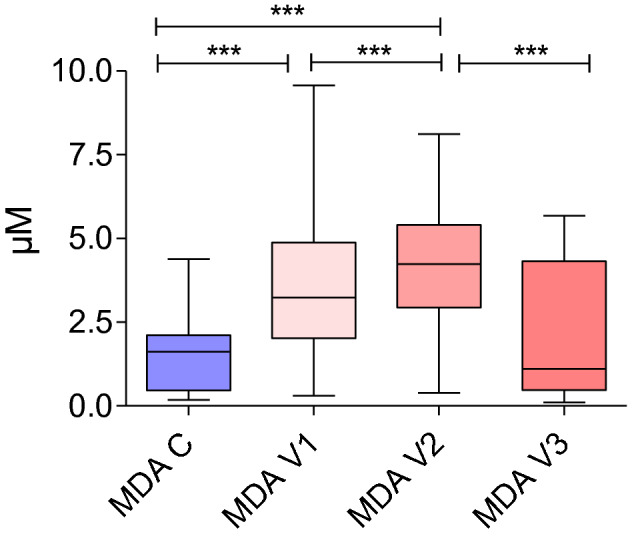
MDA concentrations in the DTC patients among the ablation treatments. ***p < 0.001; *C* control group; *MDA* malondialdehyde; *v* visit.

During the second visit (V2), performed three days after RAI, the TSH, TGB, LDL, CRP, and glucose concentrations were significantly higher in the DTC patients compared to the control group (*p* < 0.001, *p* < 0.01, *p* < 0.01, *p* < 0.01, and *p* < 0.05, respectively). Moreover, when comparing visits 1 and 2, the TSH concentration was significantly higher during visit 2 (*p* < 0.001), while CHOL, fT3, and fT4 were considerably lower (all *p* < 0.05). No difference between the TGB, TG, HDL, CHOL, and TGBAb concentrations measured between the study (V2) and control group was observed (Fig. 2). During the third visit performed one year after RAI, the TSH, LDL, CRP, and glucose concentrations remained upregulated in the DTC patients compared to the healthy subjects (*p* < 0.001, *p* < 0.05, *p* < 0.05, and *p* < 0.05, respectively). Nevertheless, the fT3, fT4, CHOL, TG, HDL, TGB and TGBAb concentrations at visit 3 showed no difference between the study and control groups (Fig. 1).

Comparing the baseline (V1) and one-year observation (V3) follow-up measurements, the concentrations of CHOL, LDL, HDL, TG, glucose, CRP, fT4, TGB and TGBAb did not significantly differ among the DTC patients. Accordingly, a significant decrease in the TSH concentration was observed in the study group compared to visit 1 (*p* < 0.01). Moreover, none of the participants was diagnosed with DTC relapse in one year observation.

### Ablation-induced effects on MDA serum profile

The MDA concentrations determined at V1 and V2 were significantly higher in the DTC patients compared to the control group (*p* < 0.001). No significant difference between V3 and the control group in the MDA measurements was observed (*p* > 0.05). Concerning the ablation scheme among the DTC patients, the MDA concentrations measured during V2 were higher compared to those obtained during V1 and V3 (both *p* < 0.001). Additionally, the MDA concentrations at visit 3 were also significantly decreased compared to V1 measurement (*p* < 0.001) (Fig. 2).

### Relationship between the oxidative stress marker and biochemical measurements

A Spearman’s rank-order regression was performed to study the relationship between MDA and both the baseline (visit 1) and follow-up (visits 2 and 3) biochemical parameters. A positive correlation was observed between baseline MDA and HDL (*r* = 0.52; *p* < 0.05) and CHOL (*r* = 0.58; *p* < 0.05). Additionally, the MDA at visit 1 was correlated with LDL (*r* = 0.32; *p* < 0.05) and CHOL (*r* = 0.45; *p* < 0.05) at visit 2, as well as TG (*r* = 0.37; *p* < 0.05), LDL (*r* = 0.40; *p* < 0.05), and CHOL (*r* = 0.33; *p* < 0.05) at visit 3.

Considering the parameters for visit 2, MDA was found to be correlated with CHOL (*r* = 0.34; *p* < 0.05) and TSH (*r* = 0.31; *p* < 0.05). However, a correlation was also observed between MDA measured three days after ablation and baseline TG (*r* = –0.51; *p* < 0.05) and HDL (*r* = 0.52; *p* < 0.05), and LDL measured one year after ablation treatment (*r* = 0.29; *p* < 0.05).

Moreover, considering visit 3 only, the MDA concentration was correlated with fT4 (*r* = 0.29; *p* < 0.05).

The Spearman’s correlations between MDA at visit 3 and fT3, LDL, and CHOL at visit 1 were *r* = 0.35, *r* = 0.51, and *r* = 0.53, respectively (*p* < 0.05 for all). The MDA at visit 3 was also correlated with fT4 (*r* = –0.32; *p* < 0.05) and CHOL (*r* = 0.30; *p* < 0.05) at visit 2.

Considering the relationship between MDA concentrations during the study and the ^131^I dose, a negative correlation was observed only in the case of MDA at visit 3 (*r* = –0.49; *p* < 0.05). The MDA measured at visit 1 was positively correlated with the MDA concentration one year after ablation (*r* = 0.29; *p* < 0.05). No relationship was observed between the MDA concentrations, CRP, and glucose (Table 2).

**Table 2:** Spearman’s correlation of the MDA concentrations and the other measurements at visit 1 (baseline), visit 2 (3 days after the RAI treatment), and visit 3 (1 year after the RAI treatment).

	MDA (V1)	MDA (V2)	MDA (V3)
MDA (V1)		0.68	0.29*
TSH (V1)	0.06	0.01	0.03
fT3 (V1)	–0.14	–0.03	0.35*
fT4 (V1)	–0.20	–0.15	0.04
TGB (V1)	0.01	−0.14	−0.15
TGBAb (V1)	–0.25	–0.16	0.01
^131^I dose	–0.06	0.04	–0.49*
CRP (V1)	–0.07	–0.1	–0.04
TG (V1)	0.00	–0.51*	–0.37
LDL (V1)	0.44	0.46	0.51*
HDL (V1)	0.52*	0.52*	0.44
CHOL (V1)	0.58*	0.39	0.53*
Glucose (V1)	0.32	0.21	0.36
MDA (V2)	0.68		0.30*
TSH (V2)	0.21	0.27*	0.26
fT3 (V2)	–0.13	–0.07	–0.06
fT4 (V2)	–0.03	–0.10	–0.32*
TGB (V2)	0.04	−0.004	−0.23
TGBAb (V2)	–0.24	–0.17	0.07
CRP (V2)	0.27	0.07	0.05
TG (V2)	0.51	0.17	0.08
LDL (V2)	0.32*	0.21	0.18
HDL (V2)	0.04	0.07	0.24
CHOL (V2)	0.45*	0.29*	0.30*
Glucose (V2)	0.04	0.0	–0.09
MDA (V3)	0.29*	0.30*	
TSH (V3)	0.02	0.04	–0.03
fT3 (V3)	–0.31	–0.24	–0.33
fT4 (V3)	–0.14	–0.07	0.29*
TGB (V3)	0.04	0.06	0.07
TGBAb (V3)	–0.29	–0.25	0.15
CRP (V3)	0.18	0.02	–0.01
TG (V3)	0.37*	0.11	–0.06
LDL (V3)	0.40*	0.29*	0.07
HDL (V3)	–0.07	0.00	0.12
CHOL (V3)	0.33*	0.18	0.12
Glucose (V3)	0.11	0.00	0.17

^*131*^*I* radioiodine, *TGBAb* thyroglobulin antibodies, *CHOL* cholesterol, *CRP* human C-reactive protein, *fT3* free triiodothyronine, *fT4* free thyroxine, *HDL* high-density lipoprotein, *LDL* low-density lipoprotein, *MDA* malondialdehyde, *TG* triglyceride, *TSH* thyroid-stimulating hormone.

*p < 0.05.

**Figure 3:**
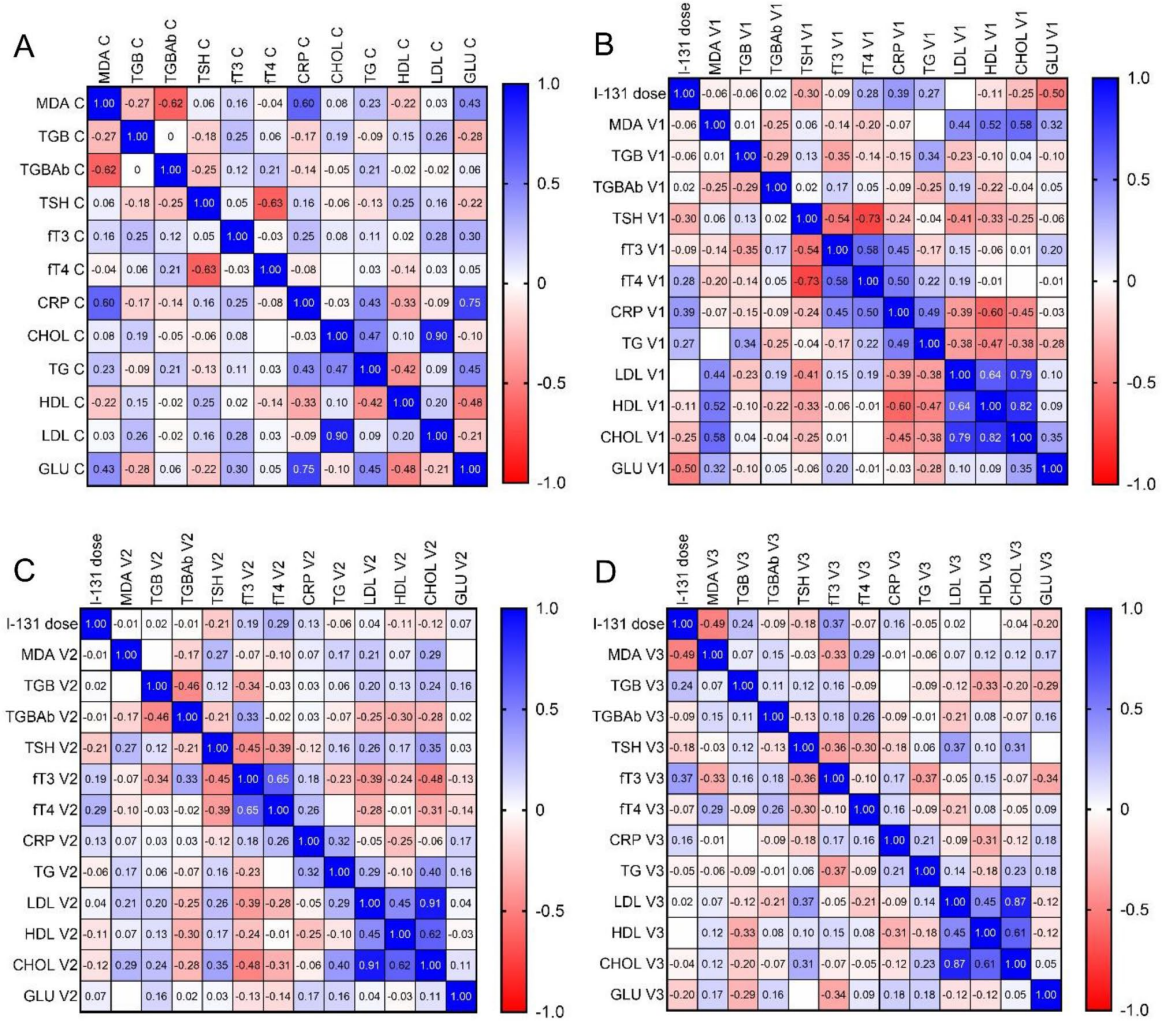
Graphical Spearman’s correlation matrix of the biochemical parameters: (**A**) control group; (**B**) visit 1 (before ablation treatment); (**C**) visit 2 (three days after ablation treatment); (**D**) visit 3 (1 year after ablation treatment); *C* control group; *CHOL* cholesterol; *CRP* C-reactive protein; *fT3* free triiodothyronine; *fT4* free thyroxine; *HDL* high-density lipoprotein; *LDL* low-density lipoprotein; *MDA* malondialdehyde; *TG* triglyceride; *TGB* thyroglobulin; *TGBAb* thyroglobulin antibodies; *TSH* thyroid-stimulating hormone; *V* visit.

Additionally, a correlation matrix was employed to evaluate the relationship between the biochemical parameters in the DTC patients. Considering the control group, a negative correlation was observed between TSH and fT4 (*r* = –0.63; *p* < 0.05) and between glucose (GLU) and HDL (*r* = –0.48; *p* < 0.05). A positive correlation between CRP and GLU was also demonstrated (*r* = 0.75; *p* < 0.05), as well as between TG and GLU (*r* = 0.45; *p* < 0.05) (Fig. 3A).

Considering the visit 1 parameters, a strong negative correlation between TSH and fT3 (*r* = –0.54; *p* < 0.05), as well as between TSH and fT4 (*r* = –0.73; *p* < 0.05), was observed. Furthermore, the medium negative correlation was observed between TGB and TGBAg (*r* = –0.29; *p* < 0.05) and between TGB and fT3 (*r* = –0.35; *p* < 0.05). Additionally, the TSH concentration correlated with the ^131^I dose in the DTC patients three days after ablation treatment. The fT4 concentration also correlated with fT3 and CRP (*r* = 0.58 and *r* = 0.50, respectively; both *p* < 0.05). A correlation between LDL and CHOL (*r* = 0.79) and HDL (*r* = 0.64) was also observed (both *p* < 0.05) (Fig. 3B).

Among the biochemical parameters measured three days after ablation, TGBAb was in correlation with fT3 (*r* = 0.33), HDL (*r* = –0.30) and TGB (*r* = –0.46) (both *p* < 0.50). Moreover, the TSH concentration correlated with fT3 (*r* = –0.45), fT4 (*r* = –0.39), and CHOL (*r* = 0.35) (all *p* < 0.05). Furthermore, medium negative correlation was observed between TGB and fT3 concentration (*r* = −0.34; *p* < 0.05). Correlations between TG and LDL, CRP, and CHOL were likewise demonstrated (*r* = 0.29, *r* = 0.32, and *r* = 0.40, respectively; all *p* < 0.05). No association between biochemical measurements and the ^131^I dose used in the ablation treatment was observed (Fig. 3C).

Considering the follow-up parameters one year after ablation treatment, fT3 correlated with TSH (*r* = –0.36), TG (*r* = –0.37), and the ^131^I dose used (*r* = 0.37) (*p* < 0.05 for all). Furthermore, medium negative correlation was observed between TGB and HDL concentration (*r* = –0.33; *p* < 0.05). A correlation was also found between TSH and LDL (*r* = 0.37; *p* < 0.05), as well as between HDL and CRP (*r* = –0.31; *p* < 0.05) (Fig. 3D).

## Discussion

Thyroid cancer is a significant clinical problem due to the growing number of patients suffering from this disease^7,9^. Surgery is the treatment of choice for primary and recurrent DTC^11^. At present, to reduce the risk of tumor recurrence after DTC resection, RAI is applied^12^. However, despite its beneficial therapeutic effect, exposure to radiation can lead to several oxidative alterations in tissue metabolism^5,13,14^. Thus, comprehensive studies are needed to demonstrate the possible health effects of DTC patients undergoing RAI treatment. Research on the influence of RAI on liver function in DTC patients showed a significant decrease in total protein albumin, globulin, alanine aminotransferase, and γ-glutamyl transferase after six months of ablation, suggesting a negative effect on liver function^15^. Moreover, Sen et al. observed that bilirubin is elevated after multiple high-dose RAI treatments, indicating the potential protective effect of bilirubin on liver function^15^. Some of the literature data supports the hypothesis that exposure to chronic oxidative stress can enhance the progression of radiation-induced late side effects, which have been analyzed in clinical studies^5,16–19^. The results of previously published studies confirmed the early redox imbalance on the third day after RAI, which is in agreement with the results obtained in our study^6^. The significant increase in the MDA concentration observed at visit 2 compared to visits 1 and 3 clearly demonstrates the early increase of oxidative stress after ablation treatment. The health effects induced by oxidative stress should be considered in DTC patients. Thus, additional antioxidant supplementation could be implemented to neutralize the increased level of reactive oxygen species (ROS) responsible for the RAI-related side-effect occurrences. Although the pathophysiology that underlies the association of RAI with oxidative stress in DTC patients requires further investigation, supplementation with antioxidants may be useful due to their radioprotective effects^20^. Notably, Jafari et al. demonstrated the radioprotective effect of vitamin C supplementation in DTC patients ablated with RAI^18^. Richter et al. observed an increase in concentrations of oxidative stress markers such as myeloperoxidase and oxidized low-density lipoproteins during the first two days after ablation^21^. A significant increase in the plasma of 8-Epi-prostaglandin F2 alpha (8-epi-PGF2a), as one of the oxidative stress markers, was observed by Rosário et al. after RAI treatment^5^. However, Makarewicz et al. demonstrated that the ^131^I DTC remnant ablation does not increase oxidative damage to membrane lipids within the first days of therapy^22^. Considering that, based on the literature data, oxidative damage to membrane lipids may also increase after more than five days, the late effects of RAI should be also established^23^. RAI may pose a long-term challenge in terms of increased oxidative stress levels. Based on their meta-analysis, Einor et al. found strong effects on oxidative status in response to low-dose ionizing radiation with a large magnitude of the mean effects^24^. Regarding to the obtained results, the lack in differences observed between the MDA concentration measured at visit 3, compared to the control group, suggests the redox stabilization in DTC patients one year after RAI therapy.

In our study, as a comprehensive approach, the long-term impact of RAI on DTC patients during ablation treatment was determined, and a significant upregulation of CRP and LDL levels in DTC patients during the study were observed. Moreover, three days after ablation, increased glucose and TSH concentrations were noted. To assess the oxidative stress level, the MDA concentrations were determined at the day of qualification for treatment, three days after RAI (performed after recombinant thyrotropin (rhTSH) stimulation), and 12 months after ablation. The MDA levels demonstrated in the control group during the analysis were comparable to those recorded by others^25,26^. We observed significant changes between the MDA concentration measured before, soon after, and one year after ablation treatment. An increase in the oxidative stress level was demonstrated during V1 and V2, resulting from the early period after ablation potentially caused by ^131^I application. The MDA concentration assessed three days after ablation was correlated with the TSH level. In this case, independent analysis of oxidative stress intensity is challenging due to the greatly increased TSH concentration observed after rhTSH application. Much of the literature data underlines the extrathyroidal effect of a high TSH level. Firstly, proangiogenic effect of microvascular endothelial cells has been suggested, i.e.TSH enhances proliferation and stimulation of capillary network formation by increased vascular endothelial growth factor (VEGF) synthesis^27–29^. Furthermore, using human microvascular endothelial cells, TSH was shown to stimulate angiogenesis and proliferation^30^. It could be hypothesized that the effects of thyroid dysfunction observed in patients with pre-existing heart failure could contribute to cardiovascular diseases^31^. Recently, it has been emphasised that TSH may be involved in anti-lipolytic/pro-lipogenic effects, because TSH could decreased adipose triglyceride lipase synthesis, and increased TG accumulation due to disturbed TG synthesis^32^. On the other hand, it was suggested that iatrogenic thyroid hormone influence could be combined with mimicking effects of paraoxonase activity, insulin sensitivity and oxidative stress, by nitrogen oxide production via the TSHR/AKT signalling pathway inhibition^33^. These findings suggest that extrathyroidal effect of high TSH level can be observed during RAI treatment^34^.

Moreover, a reduction in the oxidative stress level after one year of ablation was observed. Although a direct association between the ^131^I dose and the intensity of oxidative stress is expected in DTC patients, a positive correlation between ^131^I dose and MDA concentration after only one year after RAI was noted. In this case, MDA measurements could be used to predict the side effects of RAI. The application of ^131^I causes oxidative stress-related side effects, in this case, sensitive parameters are still needed for the screening of DTC patients^35^. Moreover, considering the limited long-term clinical data assessing the dose-dependent impact of neoplastic recurrence after ablation, it remains difficult to determine the optimal dose of RAI^36^.

More importantly, the MDA concentration measured among the ablation scheme had a positive correlation with the lipid profile assayed before the treatment. This relationship could be explained by the origin of MDA, which is one of the final products of polyunsaturated fatty acid peroxidation^37^. Considering the controversial aspects of cholesterol-dependent tumor metastasis promotion and the persistently increased levels of LDL and CRP obtained in DTC patients, further research on novel biochemical screening biomarkers and medical target discoveries would provide many beneficial outcomes^38,39^. Literature data have underlined the increased risk of coronary heart disease and ischemic stroke incidence after thyroidectomy^28,29,40,41^. In such cases, improving the evaluation of the lipid profile and the MDA concentration before ablation may be beneficial for retaining the long-term ablation effect while simultaneously calculating the risk of developing subsequent cardiovascular- related complications.

Oxidative stress is defined as the excess production of ROS relative to antioxidant defense. Because ROS have very short life span, their detection remains difficult. Nevertheless, ROS-related tissue destruction can be observed by the final products of lipid peroxidation, such as MDA. Therefore, MDA’s diagnostic utility has been suggested in many diseases, such as colorectal cancer^42^, spontaneous intracerebral hemorrhage^43^, depression^44,45^, and alcoholic hepatitis diagnosis^46^. Furthermore, MDA has also been demonstrated to be a significant independent positive predictor of the patient depth of tumor invasion and an independent positive predictor of lymph node infiltration by cancer tissue, as well as the occurrence of metastasis^42^. However, when considering common MDA measurements in clinical practice, the need for full validation data should be considered. Providing reproducible, repeatable, and valid analytical methods could result in the development of MDA-based marker panels for the diagnosis, prognosis, and therapeutic strategies of many conditions^47^. Regardless of the source, an increase in the oxidative stress level in DTC patients has been suggested as a potential risk factor for cancer progression^48^. MDA measurements can be considered a promising screening biomarker in cancer^49–51^. Furthermore, biological variations of MDA showed low individuality (II > 1.40) following literature data recommendation^52^.These results support the hypothesis that MDA measurement is useful in DTC treatment efficiency assessment, but more data in this field is needed. Thus, there is still a need to evaluate the role of oxidative stress screening biomarkers in the follow-up of RAI therapy. Besides, our results indicate a potential role of the application of oxidative stress markers in the qualification process and follow-up after RAI therapy. Moreover, the origin of the disturbed metabolic pathways caused by increased oxidative stress level combined with RAI therapy should be also thoroughly analyzed. Our study indicates that oxidative stress-related parameter measured in the DTC patients were directly related to the RAI but also to the high TSH concentration observed after rhTSH intervention. Furthermore, the direct functions of antioxidants measured in RAI treatment were not thoroughly examined^18^. It can be assumed that disturbed metabolic processes observed in patients compartment are not particularly counteracted by additional synthesis of antioxidants. Accordingly, the literature data comparing RAI treatment side-effects are still mainly retrospective and observational, with a subsequent lack of high-quality prospective randomized clinical trials. Our analysis is of great importance in understanding the role of oxidative stress in the pathophysiology of DTC and RAI treatment. Despite the validity of this study, this research should be considered as preliminary. The small sample size should be also considered as a limitation. Further research with a simultaneous comparison of biochemical parameters, follow-up markers and MDA measurement should be carried out in the future.

In conclusion, increased oxidative stress reflected by MDA measurements in DTC patients is further enhanced by RAI, but this effect is no longer observed one year after RAI. Moreover, as a preliminary assumption, MDA may be a useful indicator of oxidative stress in DTC patients after RAI therapy, but more study in this field is needed.

## Materials and methods

### Study subjects

This research was conducted during scheduled hospitalizations at the Department of Endocrinology, Diabetology, and Internal Diseases, Medical University of Bialystok, Poland. All patients were diagnosed as having papillary DTC based on clinical laboratory tests and ultrasound imaging, then confirmed by fine needle aspiration, followed by histopathological examination underwent after total resection of the thyroid gland. Fifty-five patients with DTC were enrolled in the study and their median age was 53 years (Table 3).

**Table 3:** The study and the control group characteristics.

	Study group	Control group
Number of patients	55	20
Median age (upper and lower quartiles) SE	53 (50.54; 62.52) 1.99	52 (48.24; 60.23) 2.87
Sex	M: 20	M: 8
	F: 35	F: 12
**Menopausal status**		
Premenopausal	7	3
Postmenopausal	28	9
Stage of advancement (TNM)	pT1a: 30 pT1a(m): 14 pT1b: 9 pT1b(m): 5 pT2: 6 pT3/pT4: 10	–

*F* female, *M* male, *(m)* multifocal, *p* papillary cancer, *TNM* cancer tumor-node-metastasis classification for differentiated and anaplastic thyroid cancer (based on the characteristics of primary tumor site (pT)), *pT1a* tumor size ≤ 1 cm in greatest dimension limited to the thyroid, *pT1b* tumor > 1 cm but ≤ 2 cm in greatest dimension, limited to the thyroid, *pT2* tumor size > 2 cm but ≤ 4 cm, limited to the thyroid, *pT3/pT4* tumor size > 4 cm, with gross extrathyroidal extension, *SE* standard error^53^.

A necessary sample size to detect the significant differences in all studied parameters between groups was confirmed using power analysis. Considering 5% margin of error and 95% confidence level, recommended sample size for our preliminary study is 16. Following recruitment analysis, 55 patients were enrolled to study group and 20 healthy volunteers were qualified for the control group. The study groups were homogeneous and did not differ regarding age and sex. The inclusion criteria were as follows: patients treated for DTC, no other comorbidities, no use of immunosuppressive medicaments, currently non-smokers and an overall health status assessed as good. Patients undergoing first RAI ablation 2-3 months after total thyroid resection were qualified to the study. Patient with stage pT1a were qualified to RIT based on the individual clinicists decision because of an incomplete resection of the neoplastic lesion and/or thyroid gland confirmed by imaging tests (scintigraphy and neck ultrasound). Furthermore, some patients were diagnosed with multifocal carcinoma with angioinvasion and/or capsular infiltration and had increased thyroglobulin concentration. For purpose of this study, the patients were hospitalized at three timepoints: for the qualification to treatment (V1), 3 days after RAI treatment (performed after rhTSH stimulation (V2) and 12 months after RAI treatment (V3).

The doses of radioiodine were tailored to every patient based on the size of the cancer residue and general health conditions (mean dose 3516 ± 1132 MBq; 95 ± 30.6 mCi), according to the national guidelines^54^. Ultrasound examinations were performed at every visit during the study. The control group consisted of 20 heathy volunteers with a median age of 52 years. The control group inclusion criteria were based on the lack of occurrence of any chronic disease, inflammations or the undergoing of any additional treatments to avoid interference with other pathologies. The median age of the whole group (study and control) was 55 years (quartile 25—48.5 years; quartile 75—62.5 years).

### Sample collection and measurement

Venous blood (5.5 mL) was obtained at the days of the subsequent visits and centrifuged, with serum subsequent separation and then frozen at − 80 °C. The procedures were approved by the Local Ethics Committee of the Medical University of Bialystok, Poland, and written informed consent was obtained from each participant (R-I-002/491/2019).

The TSH, free triiodothyronine (fT3), free thyroxine (fT4), and thyroglobulin antibody (TGBAb) concentrations were measured by a Roche E411 device (Roche Diagnostics, Sussex, UK) using the electrochemiluminescence (ECLIA) method. The triglyceride (TG), low-density lipoprotein (LDL), high-density lipoprotein (HDL), cholesterol (CHOL), and C-reactive protein (CRP) concentrations were assayed using the enzymatic colorimetric method on a Roche C111 device (Roche Diagnostics, Basel, Switzerland). The MDA concentration was determined using the thiobarbituric acid reactive substance (TBARS) reaction, through which MDA can be quantified calorimetrically following its controlled reaction with thiobarbituric acid (intra-assay coefficient of variation (CV)—4.9%; inter-assay CV—2.5%, limit of detection (LOD) –0.06 µM; limit of quantification (LOQ)—0.2 µM), (TBARS Assay Kit; Cayman Chemical Corp., USA, 10009055), which was performed according to the manufacturer’s recommendations. Samples and controls were measured using the blind analysis method in the same run.

### Statistical analysis

Statistical analyses were performed using GraphPad Prism 9.0 software (GraphPad Software, Inc., San Diego, CA, USA). The preliminary statistical analysis (Shapiro–Wilk test) revealed that the studied parameters did not follow a normal distribution. Thus, nonparametric tests were used for the statistical analysis between groups. All data are presented as the median and quartiles. A Mann–Whitney *U* test for independent samples was used to examine the statistical differences in the clinical parameters between the DTC and control patient samples. Additionally, a Friedman’s repeated measures analysis of variance and post-hoc Dunn’s test were performed to compare the biochemical parameters between the visits among DTC patients. Correlations were determined using nonparametric Spearman’s tests using GraphPad Prism 9.0 (GraphPad Software, Inc., San Diego, CA, USA); *p* < 0.05 was considered statistically significant.

### Institutional review board statement

The study was conducted according to the guidelines of the Declaration of Helsinki and the procedures were approved by the Local Ethics Committee of the Medical University of Bialystok, Poland (R-I-002/491/2019).

### Informed consent statement

Informed consent was obtained from all subjects involved in the study.

## Data Availability

The data presented in this study are available on request from the corresponding author. The data are not publicly available to protect personal data.
